# Interplay between swine enteric coronaviruses and host innate immune

**DOI:** 10.3389/fvets.2022.1083605

**Published:** 2022-12-22

**Authors:** Mingwei Li, Longjun Guo, Li Feng

**Affiliations:** State Key Laboratory of Veterinary Biotechnology, Harbin Veterinary Research Institute, Chinese Academy of Agricultural Sciences, Harbin, China

**Keywords:** SeCoV, apoptosis, autophagy, innate immunity, across species transmission

## Abstract

Swine enteric coronavirus (SeCoV) causes acute diarrhea, vomiting, dehydration, and high mortality in neonatal piglets, causing severe losses worldwide. SeCoV includes the following four members: transmissible gastroenteritis virus (TGEV), porcine epidemic diarrhea virus (PEDV), porcine delta coronavirus (PDCoV), and swine acute diarrhea syndrome coronavirus (SADS-CoV). Clinically, mixed infections with several SeCoVs, which are more common in global farms, cause widespread infections. It is worth noting that PDCoV has a broader host range, suggesting the risk of PDCoV transmission across species, posing a serious threat to public health and global security. Studies have begun to focus on investigating the interaction between SeCoV and its host. Here, we summarize the effects of viral proteins on apoptosis, autophagy, and innate immunity induced by SeCoV, providing a theoretical basis for an in-depth understanding of the pathogenic mechanism of coronavirus.

## 1. Introduction

### 1.1. Swine enteric coronavirus

Coronaviruses are one of the most devastating pathogens. The sudden outbreak of COVID-19 in 2019 has had a major impact on global public health and economic development, while the devastating effects of CoVs are not only limited to humans but also occur in livestock populations. Swine enteric coronaviruses (SeCoV) pose a huge threat to the global farm industry. Four SeCoVs were identified: porcine epidemic diarrhea virus (PEDV), porcine transmissible gastroenteritis virus (TGEV), porcine delta coronavirus (PDCoV), and swine acute diarrhea syndrome coronavirus (SADS-CoV). In the last century, PEDV and TGEV were first reported (1–3), and then widely spread to many swine-producing countries in Europe and Asia (4, 5). Recently, PDCoV and SADS-CoV have emerged as SeCoVs (6, 7), Compared with PEDV and TGEV, the clinical signs caused by PDCoV and SADS-CoV infection are less severe, and the mortality rate of newborn piglets is 30–40%. It is worth mentioning that in 2021, US scientists discovered that the plasma samples of three Haitian children with unexplained fever tested positive for PDCoV, in which suggesting the risk of PDCoV across species transmission (8).

**Figure 1:**
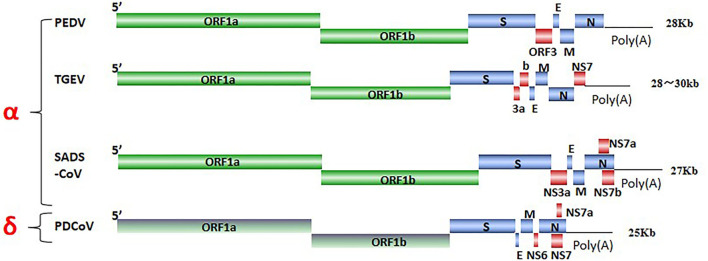
The genome structures of SeCoV.

SeCoV is a single-stranded, positive-sense RNA virus, and its viral genome consists of structural proteins, non-structural proteins, and accessory proteins. Four structural proteins, spike (S), envelope (E), membrane (M), and nucleocapsid (N) proteins were identified. The S protein mediates attachment to the host receptor and is a trimer with an S1 subunit that contains the large receptor-binding domain (RBD) and an S2 subunit that contains peptides mediating cell fusion (9). E and M proteins are responsible for maintaining the structure and size of the viral envelope (10). The N protein constitutes the only protein present in the nucleocapsid and wraps the virus genome to form a nucleoprotein complex (11). ORF1ab encodes non-structural proteins *via* nsp3 and nsp5 cleavage, there are 15–16 functional non-structural proteins (nsps), These nsps are involved in the replication and transcription of viral RNA and some nsps also inhibit the host immune response (10, 12), ORF3, NS6, and NS7 encode accessory proteins to modulate viral pathogenicity (13) (Figure 1). SeCoV has become a major cause of lethal watery diarrhea in newborn piglets, imposing enormous economic losses and a public health burden on the swine industry worldwide.

### 1.2. Autophagy induce by SeCoV infection

**Figure 2:**
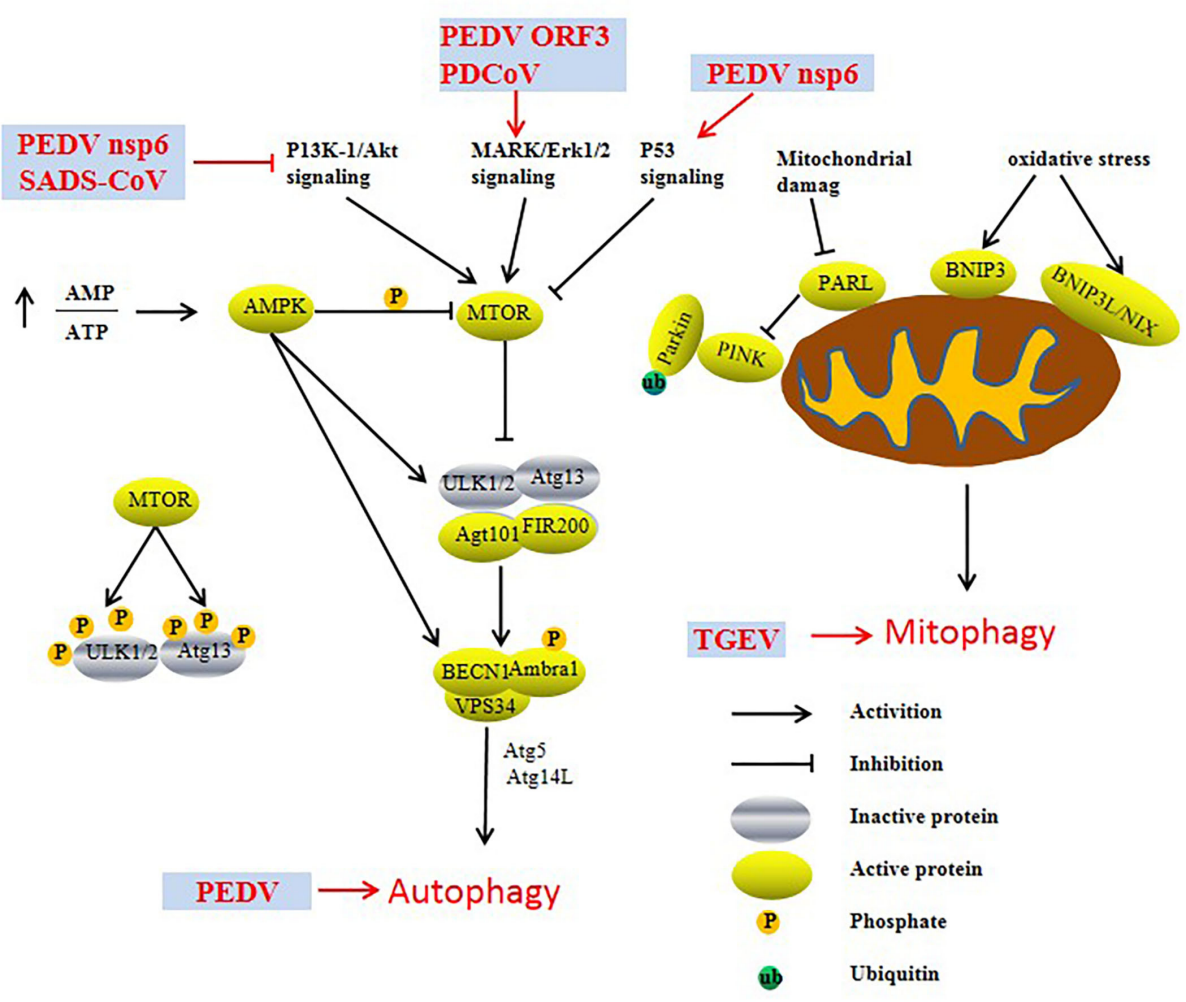
Induction and modulation of host autophagy by SeCoV infection.

Autophagy is a process in which cells use lysosomes to degrade damaged organelles and macromolecular substances under the regulation of autophagy-related genes (Atg). Previous studies have reported that autophagy is an intrinsic host defense mechanism that mediates the autophagic elimination of viral constituents or virions by targeting virus particles or virus component degradation to facilitate host innate and adaptive immunity. Increasing evidence indicates that viruses have evolved various complex strategies to escape or subvert the antiviral effects of autophagy (Figure 2). For example, SARS-CoV-2 ORF3a promotes the induction of autophagy *via* the classic ATF6 and IRE1-XBP1 UPR pathways to protect the virus from hydrolysis (14). Furthermore, ORF10 and M of SARS-CoV-2 promote the accumulation of LC3 in mitochondria and induce mitophagy, which inhibits RIG-MAVS-triggered IFN signaling (15, 16). PEDV-triggered autophagy in Vero cells *via* both the PERK and IER1 pathways promotes viral replication (17–19). Additionally, nsp6 and ORF3 of PEDV were able to induce significant autophagy in IPEC-J2 cells, and nsp6 of PEDV induced autophagy by inhibiting the PI3K/Akt/mTOR signaling pathway, which promotes cell damage and enhances the virulence of PEDV (20). Moreover, PEDV ORF3 protein triggers the endoplasmic reticulum (ER) stress response by upregulating the expression of GRP78 protein and activating the PERK-eIF2α signaling pathway to induce autophagy (21). In addition, SADS-CoV and PDCoV induce autophagy to facilitate viral replication *via* the PI3K/Akt/mTOR signaling pathway *in vitro* (22–24).

TGEV infection induces mitophagy to suppress oxidative stress and apoptosis in porcine epithelial cells (IPEC-J2 cells) to promote cell survival and, possibly, viral infection. Furthermore, N of TGEV may be involved in mitochondrial damage and mitophagy induction during TGEV infection (25). Interestingly, TGEV infection activates autophagy, whereas autophagy inhibits TGEV replication (26). Upon PDCoV infection, the upregulation of the LC3-II/LC3-I ratio and the downregulation of p62 protein levels indicate that PDCoV infection may induce autophagy, similar to other CoVs (22, 27). Additionally, PDCoV-induced autophagy enhances viral replication through the p38 signaling pathway (28).

### 1.3. Apoptosis induce by SeCoV infection

Apoptosis refers to the autonomous and orderly death of cells that is controlled by genes to maintain the stability of the internal environment. It is involved in the activation, expression, and regulation of a series of genes. There are two pathways of apoptosis, extrinsic and intrinsic. The extrinsic apoptotic pathway is mediated by death receptors (DRs) on the cell membrane, thereby activating the cascade of apoptosis signaling pathways. The intrinsic apoptosis pathway is mainly activated by apoptosis inducers in the cytoplasm to activate mitochondrial pro-apoptotic factors and destroy the integrity of the mitochondrial outer membrane. Subsequently, the increase in mitochondrial outer membrane permeability (MOMP) promotes the release of cytochrome c (Cyt c) and apoptosis-inducing factor (AIF), thereby inducing apoptosis (29).

**Figure 3:**
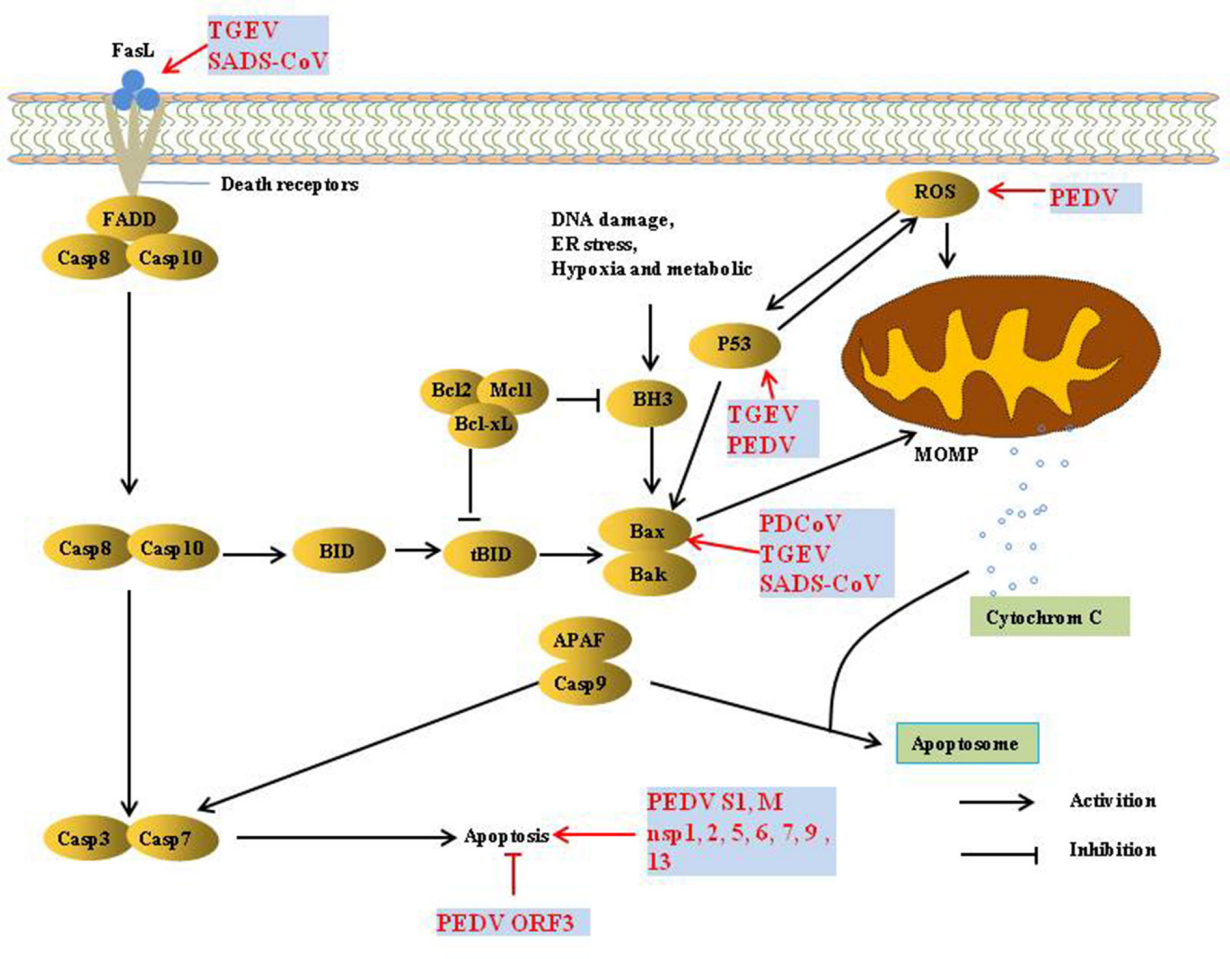
Induction and modulation of host apoptosis by SeCoV infection.

Apoptosis is considered a host innate defense mechanism that disrupts viral replication by eliminating virus-infected cells, but some viruses utilize apoptosis as a mechanism for cell killing and viral spread (30) (Figure 3). PEDV infection induces apoptosis *via* a caspase-independent mitochondrial AIF-mediated pathway to facilitate viral replication (31–33). It has been demonstrated that the activation of p38 MAPK and JNK cascades also contributes to PEDV replication, but they are not linked to PEDV-mediated apoptosis (34, 35). P53 plays an essential role in viral infection-induced apoptosis. PEDV and TGEV induce apoptosis *via* a P53-dependent pathway (35, 36). PEDV infection activated P53-puma and reactive oxygen species (ROS)/p53 signaling pathways to induce apoptosis in Vero cells and cause cell cycle arrest at the G0/G1 phase (35, 37). The S1 protein of many coronaviruses can induce cell apoptosis; the PEDV S1 protein is the main inducer of cell apoptosis during PEDV infection, and PEDV M and nsp1, 2, 5, 6, 7, 9, and 13 also induce cell apoptosis, but to a lesser extent. Similarly, the S protein of TGEV can strongly induce apoptosis in Vero-E6 cells, suggesting that S1 is a promising strategy to inhibit coronavirus infection (38, 39). In contrast, reverse genetics technology has been used to prove that the PEDV ORF3 protein promotes virus proliferation by inhibiting cell apoptosis (40).

TGEV induced apoptosis in PK-15 and ST cells but not in intestinal epithelial cells (41, 42), p53- and ROS-mediated AIF pathways, and caspase-dependent pathways both played a dominant role in triggering apoptosis. However, p38 MAPK signaling was only partially responsible for the activation of p53 and contributed less to TGEV-induced apoptosis (36, 42–45). Interestingly, the TGEV N protein is cleaved by caspase-6 and−7 during TGEV-induced apoptosis (46). However, TGEV N upregulated p53 and p21 and arrested the cell cycle at the S and G2/M phases, finally resulting in apoptosis of PK-15 cells (47).

PDCoV induces apoptosis to promote viral replication in both LLC-PK and ST cells but not in infected intestinal enterocytes *in vivo* (48). In addition, PDCoV and SADS-CoV infection induces apoptosis by recruiting Bax or opening the mitochondrial permeability transition pore (MPTP) and then releasing Cyt c, sequentially activating initiator caspase-9 and downstream effector caspase-3, thereby orchestrating the final apoptotic response to facilitate viral replication *in vitro*, Intrinsic caspase-9 dependent apoptosis pathway plays an important role in the successful replication of PDCoV and SADS-CoV (49, 50). Further studies showed that caspase-dependent FASL-mediated apoptotic pathways are also involved in SADS-CoV infection (50).

### 1.4. Innate immunity recognition of SeCoV

#### 1.4.1. Pattern recognition receptors

The innate immune response is the host's first line of defense against pathogens. Innate immune cells recognize pathogen-associated molecular patterns (PAMP) by expressing pattern-recognition receptors (PRRs), including Toll-like receptors (TLRs), RIG-I-like receptors (RLRs), NOD-like receptors (NLRs), AIM2-like receptors (ALRs), C-type lectin receptors (CLRs), and intracellular DNA sensors, such as cyclic GMP-AMP synthase (cGAS), which are key innate immune components that recognize viral components such as viral nucleic acids and proteins (51). Among these receptors, TLRs and RLRs are the two major receptors responsible for sensing RNA virus infections and triggering antiviral IFN programs. The TLR family comprises 10 members (TLR1–TLR10) in humans and 12 (TLR1–TLR9 and TLR11–TLR13) in mice (52). TLR1, TLR2, TLR4, TLR5, and TLR6 play pivotal roles in viral protein recognition (53). The membrane proteins TLR3, TLR7/8, and TLR9 are used, respectively (54–56). The RLR family includes three members: retinoic acid-inducible gene I (RIG-I), melanoma differentiation-associated protein 5 (MDA5), and laboratory of genetics and physiology 2 (LGP2). RIG-I and MDA5 are activated by immunostimulatory RNA, which leads to the activation of cytoplasmic kinases that promote the activation of interferon regulatory factor 3 (IRF3), IRF7, and nuclear factor-kappa B (NF-κB). Activated IRF3/IRF7 binds to PRD I/III sequences and induces the expression of type I IFN genes (57). The activated form of NF-κB translocates to the nucleus and triggers IFN-β expression by binding to PRD II elements (58). IFN is then secreted, which binds to receptors on virus-infected cells, as well as uninfected neighboring cells, and activates the JAK/STAT pathway to generate hundreds of ISGs to establish an antiviral state (59).

#### 1.4.2. Immune evasion mechanisms of SeCoV

**Figure 4:**
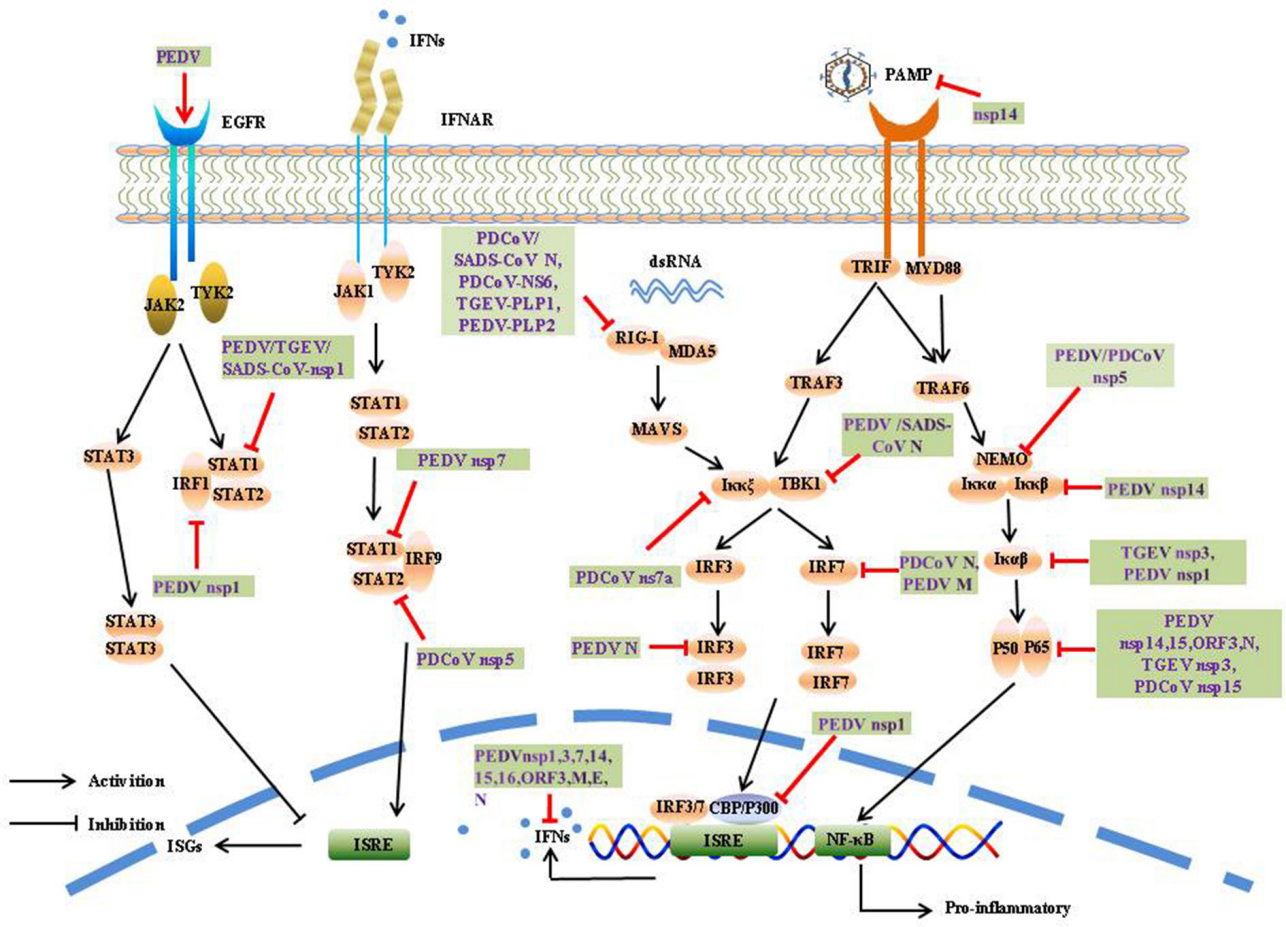
Innate immunity and modulatory mechanisms during SeCoV infection.

The induction of IFN-α/β is the most rapid and effective mechanism by which the host initiates innate immune responses. To counter innate immune signaling, many coronaviruses have evolved different strategies to develop multiple strategies to evade the innate immune response and efficiently promote their replication and infective capacity, particularly by minimizing IFN production and inhibiting IFN signaling (Figure 4).

##### 1.4.2.1. Ubiquitination and deubiquitination induced by SeCoV

Ubiquitination is a critical biological process in the post-translational modification of proteins and involves multiple signaling pathways such as protein metabolism, apoptosis, DNA damage, cell-cycle progression, and cancer development. Ubiquitin is mainly connected in eight ways (M1, K6, K11, K27, K29, K33, K48, and K63), and can regulate different functions of substrate proteins. For example, K48 polyubiquitination mainly plays the role of the ubiquitin-proteasome system to degrade substrates and proteins, whereas K63 polyubiquitination mainly regulates endocytosis, protein interaction, and signal transduction (60).

The CoV N protein, the most abundant viral protein, plays a key role in IFN interruption. The SADS-CoV N protein mediates K27-, K48-, and K63-linked ubiquitination of RIG-I and its subsequent proteasome-dependent degradation to inhibit the host IFN response (61). The PDCoV N protein could directly target porcine RIG-I to interfere with its binding to dsRNA and block its early activation by blocking porcine Riplet (pRiplet)-mediated K63-linked polyubiquitination, thus suppressing IFN-β Production (62). In addition, PDCoV N protein promotes poIRF7 degradation through the K6, K11, and K29 polyubiquitination-proteasome pathways to reduce type-I IFN production (63).

The deubiquitinase (DUB) family is responsible for the specific hydrolysis of ubiquitin molecules from ubiquitin-linked proteins or precursor proteolysis, which affects the localization, stability, and function of target proteins in cells, DUB are widely present in various viruses, and significantly influences viral activity. Interestingly, all CoV DUB activities are mediated by PLPs, and the PLPs of human coronavirus NL63 (HCoV-NL63), SARS-CoV, MHV, and MERS-CoV significantly reduced the levels of ubiquitinated STING, RIG-I, TBK1, and IRF-3, thereby negatively affecting the regulation of host antiviral innate immunity (64). Likewise, PEDV PLP2 and TGEV PL1 strongly inhibit RIG-I- and STING-activated IFN expression *via* deubiquitination (65, 66). A recent study showed that SADS-CoV PLP2 could also function as a DUB, such as PEDV PLP2, SARS-CoV PLpro, and TGEV PLP1 (67).

##### 1.4.2.2. Protein cleavage

SeCoV encodes non-structural protein 5, also called the 3C-like protease, and is responsible for coronavirus polyprotein processing. It cleaves the polyprotein at more than 11 sites to yield the essential proteins required for virus replication and pathogenesis. At the same time, the protease can also use its cleavage activity to cleave host proteins, especially the key molecules of IFN production and signal transduction, and play an immunomodulatory role. 3CLpro is an attractive drug target because it is highly conserved among known coronavirus species. Many viruses antagonize innate immune signaling by cleaving 3C-like proteases; for example, porcine sapelovirus (PSV) 3Cpro inhibits the production of IFN-β by cleaving MAVS and degrading MDA5 and TBK1 (68). Enterovirus 71 (EV71) 3C interacts with and cleaves TAB2 and TAK1 to interfere with the inflammatory responses (69). Norovirus (NoV) encoding a 3C-like protease was found to effectively suppress Sendai virus (SEV)-mediated IFN-β production by cleaving the NF-κB essential modulator (NEMO) (70). Similar to NoV and EV71 3Cpro, PEDV and PDCoV encode a 3C-like protease, nsp5, which is an IFN antagonist that cleaves NEMO at Q231, suggesting that NEMO may be a common target for coronaviruses (71, 72). In addition, PDCoV nsp5 also suppressed IFN signaling by cleaving STAT2, a key molecule in the JAK-STAT pathway, nsp5 cleaved STAT2 at both Q685 and Q758 (73).

##### 1.4.2.3. Competitive binding

It has been demonstrated PEDV N protein directly interacts with TBK1, thereby sequestering the association between TBK1 and IRF3, which in turn inhibits both IRF3 activation and type I IFN production (74). Moreover, the PDCoV N protein antagonizes IFN-β production by interfering with the binding of dsRNA and protein activator of protein kinase R (PACT) to RIG-I (75). The SADS-CoV N protein suppresses the RLRs Signaling pathway. Moreover, the SADS-CoV N Protein not only blocked the IPS-1–TBK1 interaction but also disrupted the formation of the TNF receptor-associated factor 3(TRAF3)–TBK1 complex, which led to reduced TBK1 activation and IFN-β production (76). It has been demonstrated PEDV nsp7 antagonizes type I IFN production, PEDV nsp7 also antagonized IFN-α-induced JAK-STAT signaling by sequestering the interaction between karyopherin α1 (KPNA1) and STAT1 (77). Cytoplasmic stress granules (SGs) can effectively exert antiviral functions; however, nsp15s of PEDV, TGEV, SARS-CoV, and SARS-CoV-2 have conserved functions that interfere with chemically induced SGs formation (78). Coronavirus accessory proteins are species specific and have low homology with other known proteins. Although current research has shown that coronavirus accessory proteins are not necessary for virus replication (79), extensive reports have indicated that many accessory proteins are involved in immune regulation and virus virulence. For example, PDCoV NS6 interacts with the CTD of RIG-I and the Hel and CTD of MDA5, and this interaction attenuates the binding of RIG-I/MDA5-dsRNA, resulting in a reduction in IFN-β production (80). PDCoV NS7a interacts with IKKε, which significantly disrupts the interaction between IKKε and TRAF3 or IRF3, thereby inhibiting IFN-β production (81).

##### 1.4.2.4. Impair phosphorylation or suppressed the nuclear translocation

Post-translational modification of proteins is a critical way to regulate protein function. Phosphorylation is one of the most extensively investigated post-translational modifications involved in the regulation of signal transduction, but viral encoded proteins can regulate phosphorylation and dephosphorylation to promote proliferation. The PEDV E protein has been found to block the production of IFN, but little is known about the process by which the E protein subverts host innate immunity. A previous study showed that PEDV E protein is responsible for inducing ER stress through activation of the PERK/eIF2α branch and activation of NF-κB (82). Further studies showed that PEDV E protein remarkably suppressed IFN-β production by interfering with the translocation of IRF3 from the cytoplasm to the nucleus through direct interaction with IRF3 (83, 84). PEDV N protein blocks NF-κB nuclear translocation to antagonize IFN-λ production (85). The PEDV M protein plays an important role in viral assembly, viral budding, and host immune mediation. PEDV M protein interacts with IRF7 and significantly suppresses its phosphorylation and dimerization of IRF7, leading to decreased expression of type I IFN (86). Existing study has been identified the sole accessory protein ORF3 of PEDV as NF-κB antagonist, it inhibits the phosphorylation of IκBα, in addition, PEDV ORF3 inhibits NF-κB activation by interfering the phosphorylation and expression of p65, as well as interfering nuclear translocation of p65, which ultimately leaded to the inhibition of IL-6 and IL-8 production (87). As a key virulence factor for coronaviruses, nsp1 impedes host protein expression *via* multiple mechanisms. Of the 21 PEDV proteins, nsp1, nsp3, nsp5, nsp7, nsp14, nsp15, nsp16, ORF3, and E inhibited NF-κB activity, and nsp1 appeared to be the most potent inhibitor. Nsp1 interfered with the phosphorylation and degradation of IκBα, and thus blocked p65 nuclear transport; however, PEDV nsp1 did not interfere with IRF3 phosphorylation and nuclear translocation, which interrupted the enhanceosome assembly of IRF3 and CREB-binding protein (CBP) by degrading CBP, resulting in the inhibition of ISGs expression (88, 89). Furthermore, nsp1 was found to suppress type III IFN activity by blocking the nuclear translocation of interferon regulatory factor 1 (IRF1) and reducing the number of peroxisomes (90). PEDV, TGEV, and SARS-CoV nsp1 significantly inhibited the phosphorylation of STAT1 at S727, interfering with the effect of IFN-I, and nsp1 also arrested host cells to stay in the G0/G1 phase (91, 92). In contrast, PEDV nsp1 inhibited CCAAT/enhancer-binding protein β (C/EBP-β) phosphorylation to reduce complement component 3 (C3) expression, which is considered to play a crucial role in preventing viral infection (93). Nsp14 of CoV has ExoN and guanine-N7-methyltransferase (N7-MTase) activities (94, 95), playing a key role in viral mRNA cap synthesis, CoV replication and transcription, Howerver, the function and mechanism by which nsp14 modulates and manipulates host immune responses remain largely unknown, Recently study showed PEDV nsp14 remarkably decreased NF-κB activation and proinflammatory cytokines expression, it interacted with Iκκs and p65 to inhibite the phosphorylation of Iκκs. Furthermore, nsp14 suppresses TNF-α-induced phosphorylation and nuclear import of p65 (96). TGEV nsp3 has been shown to strongly inhibits NF-κB signaling by suppressing Iκ*βα* degradation and inhibiting p65 phosphorylation and nuclear translocation (97). Nsp15 encodes an endoribonuclease that conserves all coronaviruses. The nuclease activity of nsp15 plays a critical role in viral evasion by triggering an innate immune response. PDCoV nsp15 significantly inhibits IFN-β production by disrupting the phosphorylation and nuclear translocation of p65, independent of its endoribonuclease (98). TGEV ORF7 binds to the catalytic subunit of protein phosphatase 1 (PP1c) and regulated the dephosphorylation of eIF2α to counteract host cellular defenses (99). In addition, deletion of ORF7 increased innate immune responses and acute tissue damage, demonstrating antagonism from the opposite perspective (100).

##### 1.4.2.5. Degradation and inactivation induced by SeCoV

In addition, SeCoV can antagonize the host innate immune response through degradation and inactivation. PEDV suppresses type I interferon response by stimulating epidermal growth factor receptor (EGFR) activation, which is responsible for STAT3 expression (101). PEDV nsp15 directly degrades the mRNA of TBK1 and IRF3 depending on its EndoU activity to inhibit the production of IFN and ISG and antagonize the host innate response to promote replication (102). CoV nsp14 can degrade dsRNA PAMPs to prevent IFN induction during CoV infection (103). Of the 21 PEDV proteins, nsp1, nsp3, nsp7, nsp14, nsp15, and nsp16 were found to inhibit IFN-β and IRF3 promoter activity (89). Further studies showed that nsp1, nsp3, nsp5, nsp8, nsp14, nsp15, nsp16, ORF3, E, M, and N suppressed type III IFN activity (90).

## 2. Discussion

SeCoV is a pathogenic microorganism that seriously threatens the pig industry and causes massive economic loss. The above evidence reveals the viral immune evasion mechanisms of SeCoV, where the origin of SeCoV and the interaction between the virus and host need to be further elucidated. Furthermore, the rapid global spread of highly pathogenic of SARS-CoV, MERS-CoV, and SADS-CoV-2 pose a concern about cross-species transmission, such as the discovery of PDCoV in Haitian children. It is evident that proper surveillance of viral biodiversity can be used to prevent animals becoming mixers and intermediate hosts of various coronaviruses in the future. Morever, an important feature of the epidemiology of SECoV is the emergence of several different variants, which vary in their transmissibility, virulence, clinical disease presentation, and vaccines response, resulting in unforeseeable epidemic scope and pathogenicity. Up to now, porcine aminopeptidase N (pAPN) has been identified as a receptor for TGEV, but the receptors of PEDV, PDCoV, and SASD-CoV remain unknown, hindering the development of vaccines and drugs.

Exploration of these programs will help us further understand how SeCoV exists to ensure their survival, and also provide us with new ideas for developing drug targets for the prevention and treatment of SeCoV.

## Author contributions

ML wrote the first draft of the manuscript. LG and LF contributed to conception and design of the review. All authors contributed to manuscript revision, read, and approved the submitted version.
